# Meta-Analysis: Association Between Hypoglycemia and Serious Adverse Events in Older Patients Treated With Glucose-Lowering Agents

**DOI:** 10.3389/fendo.2021.571568

**Published:** 2021-03-08

**Authors:** Katharina Mattishent, Yoon K. Loke

**Affiliations:** Norwich Medical School, University of East Anglia, Norwich, United Kingdom

**Keywords:** hypoglycemia, older people, diabetes, adverse effects, meta-analysis

## Abstract

**Aims:**

We conducted a meta-analysis of serious adverse events (dementia, macro- and micro-vascular events, falls and fractures, and death) associated with hypoglycemia in older patients treated with glucose lowering drugs.

**Materials and Methods:**

Meta-analysis of studies reporting on hypoglycemia and adverse events. The search included studies from two previously published systematic reviews, and an updated search of MEDLINE and EMBASE from April 2014 to November 2019. We assessed study validity based on ascertainment of hypoglycemia, adverse events and adjustment for confounders, and conducted a random effects meta-analyses, assessing heterogeneity using the I^2^ statistic.

**Results:**

We included 44 studies involving 2,507,434 participants. Most of the studies used adjusted analysis for confounders and hypoglycaemic events were typically identified based on healthcare databases (severe events). Hypoglycemia was associated with increased likelihood of death in a meta-analysis of eighteen studies, pooled OR 2.02 (95% Confidence Interval 1.75–2.32). Studies assessing mortality signal a time-response relationship with a higher risk of adverse events occurring within the first 90 days after hypoglycemia. Our meta-analysis of nine studies demonstrated that hypoglycaemic episodes were associated with dementia – pooled OR 1.50 (95% CI 1.29–1.74). Our meta-analysis of nineteen studies demonstrated associations between hypoglycaemia and macrovascular complications, pooled OR 1.81 (95% CI 1.70–1.94), and microvascular complications (two studies) pooled OR 1.77 (95% CI 1.49–2.10). There is also an association between hypoglycemia and cardiovascular death (six studies) – pooled OR 2.11 (95% CI 1.55 to 2.87). Similarly, our meta-analysis of six studies demonstrated an association between hypoglycemia and falls and fractures, pooled OR 1.78 (95% CI 1.44–2.21) and 1.68 (95% CI 1.37–2.07) respectively.

**Conclusion:**

This meta-analysis confirms previously reported concerns of serious harm following hypoglycemia, especially in the immediate time period after a hypoglycaemic event. Avoidance of hypoglycaemic episodes should be a priority in this vulnerable population.

## Introduction

We have previously published two meta-analyses on adverse events (dementia, macro- and micro-vascular events, falls and fractures, and death associated with hypoglycemia in older people treated with glucose-lowering drugs (1, 2). However, since those publications in 2016, we are aware of many new studies [including the authors’ own work (3)] on adverse events associated with hypoglycemia. Hence, we have updated our meta-analyses and present a comprehensive review of the up to date evidence regarding the association between hypoglycemia and adverse events in older people.

Our first systematic review looked at the bi-directional relationship between hypoglycemia and dementia (2). The key findings of the meta-analyses were a 70% increased risk of deterioration in cognition following hypoglycemia and conversely a 60% increased risk of hypoglycemia in older people with dementia. However, this review did not include other major adverse events that may be associated with hypoglycemia. Hence, our second systematic review which focused on vascular adverse events, falls and fractures and all-cause mortality. We found a 1.5 times increased risk in macrovascular events (ischaemic strokes, myocardial infarctions) and a doubling of risk in falls, fractures, and all-cause mortality, but there was insufficient data on cardiovascular death to enable meta-analysis (1). This second review also did not find any studies that specifically looked at the effects of hypoglycemia in older patients who also have dementia.

This updated search of the most recent evidence allows us to access a far larger data set and perform meta-analyses of additional cardiovascular outcomes and time-relationships that we could not previously address due to insufficient data.

## Materials and Methods

We worked from the methods described in previously published meta-analyses (1, 2).

### Data Sources and Searches

The population we were interested in was older adults. The intervention was ‘hypoglycemia’ and the comparator ‘no hypoglycemia’. The outcomes of interest were cardiovascular events, falls and fractures, death and dementia.

The searches we ran only included terms for the population and the intervention, as the outcomes are too diverse and non-specific for us to be confident that we would capture all the relevant papers if we focused on particular outcomes. For instance, myocardial infarction could be described under a multitude of terms as acute coronary syndrome, STEMI or NSTEMI.

Three searches fed into this systematic review and meta-analysis.

For both previously published reviews, we searched MEDLINE and EMBASE for a ten-year period up to March 2015 with English language restriction.

Bibliographies of included studies were checked for other potentially suitable studies. We were notified through PubMed automated updates of any new relevant studies.

The search strategy for the current review was conducted up to November 2019 and can be found in **Appendix 1**.

### Study Selection

In our analysis, we included cohort studies (prospective and retrospective), which examined the association between hypoglycemia and serious adverse events in participants aged 55 years and older on glucose-lowering medications. We used an arbitrary cut-off of 55 years because there is no internationally accepted definition, and we aimed to be broad rather than too restrictive. We treated post-hoc analyses of randomized controlled trials as cohort studies, as the analysis is no longer on a prospective randomized basis due to post-hoc classification of patients (with and without hypoglycemia).

We excluded cross-sectional studies, because it would be impossible to determine whether the intervention (hypoglycemia) or outcome (adverse events) occurred first.

We included only full journal publications because abstracts are limited in word count and cannot fully describe the statistical models and confounding variables that are of key interest in non-randomized studies.

### Data Extraction and Quality Assessment

Two authors conducted study screening and data extraction independently. Uncertainties and discrepancies were resolved through discussion.

We used a standardized form to collect data on study design, study year, geographical location, setting, selection criteria, participants’ characteristics and outcome measures. We extracted relative measures of effect such as odds ratios, risk ratios, and hazard ratios for the outcomes of interest in the group with hypoglycemia as compared to the controls (please refer to **Tables 1** and **2**). For the sensitivity analysis of survival at different time-points we have focussed only on studies that report hazard ratios.

**Table 1:** Study design and characteristics.

Study ID	Design	Data source Number of patients, setting, dates	How were the patients selected for study	Diabetes definition & Patient Characteristics, age, sex (or Selection of Cases and Controls)	Type of glucose lowering agents
Bedenis et al. (4)	Prospective cohort	Edinburgh type 2 Diabetes Study, 1066 participants, Lothian Region Scotland 2006-2010	Lothian Diabetes Register	Type 2 diabetes, mean age 68 years, male 51% 31% previous coronary heart event, 8.7% previous cerebrovascular event, 14% previous MI, 5.8% previous stroke	Oral hypoglycaemic agents, insulin
Bonds et al. (5)	Post hoc analysis of RCT	10,251 77 clinical centres in North America	RCT (ACCORD) in patients with Type 2	Type 2 diabetes, mean age 62.2 years, male 62%. Had either established cardiovascular disease or additional cardiovascular risk factors. Exclusions: past severe hypoglycaemia, BMI >45, serum creatinine >133 micromol/L, other serious illness	Insulin, oral hypoglycaemic agents
Cha et al. (6)	Prospective cohort	Vincent Type 2 Diabetes Registry enrolled between January 2000 to December 2010 (follow-up until May 2015), 1260 participants, South Korea	Vincent Type 2 Diabetes Registry	Type 2 diabetes, mean age 55 years, female 59% Exclusions: older than 75 years, mental health illness, unable to undertake self-care, previous episodes of SH, cognitive impairment, alcohol excess, malignancy, end-stage renal disease, severe infection, liver cirrhosis	Insulin, oral hypoglycaemic agents
Chiba et al. (7)	Retrospective	211 Tokyo, Japan, Dec 2009-Apr 2011	Outpatient diabetes clinic attended for ≥one year.	>60 years 168 T2DM patients and 43 age-matched, non-diabetic controls Exclusion: blindness, wheelchair/bedridden, end-stage renal disease, adrenal insufficiency, hypopituitarism, hypo/hyperthyroid, uncontrolled hypertension	Oral hypoglycaemic agents Insulin
Chin et al. (8)	Prospective cohort	Korea National Diabetes Program, 1957 participants, Korea 2006-2014	Korea National Diabetes Program database and Health Insurance Review and Assessment Service of Korea (HIRAS)	Type 2 diabetes, mean age 68 years, 47% male, mean diabetes duration 8 years Exclusions: history of hypoglycaemia. cognitive impairment, previous history of drug misuse, head injury, depression.	Oral hypoglycaemic agents insulin
Cukierman-Yaffe et al. (9)	Post-hoc analysis form ORIGIN trial (RCT)	11,495 participants recruited between 2003 to 2005, 573 sites in 40 countries	ORIGIN trial	Individuals with impaired fasting glucose, impaired glucose tolerance or early type 2 diabetes who also had additional cardiovascular risk factors Mean age 66 years (SH), 63 years (non-SH), female 26% (SH), 33% (non-SH), baseline MMSE >24 Median follow-up 6.2 years	Oral hypoglycaemic agents insulin
Davis et al. (10)	Post-hoc analysis of Veterans Affairs Diabetes Trial (VADT)	17191 military veterans 20 Veterans Administration Hospitals across the United States	VADT trial	Type 2 diabetes, 97% male, median follow-up 5.6 years, inadequate response to maximal doses of oral agents or insulin therapy. Mean age 60.4 years Exclusions: HbA1c <7.5%, cardiovascular event during previous 6 months, advanced congestive heart failure, severe angina, life expectancy <7 years, BMI>40, serum creatinine level >141 micromol/L, alanine aminotransferase level > three times the upper limit of normal	Oral hypoglycaemic agents insulin
Duckworth et al. (11)	Posthoc analysis of RCT	1791 US 1 December 2000–30 May 2008	RCT (VADT)	Mean age 60.3 years 97% male T2DM Exclusions: recent cardiovascular event, serious co-morbidities, renal or liver impairment, BMI>40	Insulin, oral hypoglycaemic agents
Escalada et al. (12)	Retrospective cohort	Medicare Advantage claims database 31035 patients 1 January 2007 to 31 December 2012 US	Medicare Advantage claims database	Patients with type 2 diabetes making first pharmacy claim for basal insulin, included if previously on GLP-1 analogs/oral hypoglycaemic agentes and had at least 2 years of Medicare Advantage coverage. Mean age 72 years. Excluded: previous insulin use (prandial insulin use during follow-up was permitted)	Oral hypoglycaemic agentes, insulin
Freemantle et al. (13)	Post hoc analysis of CREDIT study (non-interventional longitudinal study)	3601 participants enrolled between 4 December 2006 and 20 April 2008 314 centres in 12 countries	CREDIT study	inclusion criteria: Type 2 diabetes, age >40 years, started insulin >1 month and <6 months prior to study entry and who had HbA1c measurement within 3 months prior to starting insulin. Mean age 62 years, diabetes duration 11 years, type 2 diabetes	Oral hypoglycaemic agents, insulin
Goto et al. (14)	Retrospective cohort	Health insurance database 58223 patients Japan	Japan Medical Data Centre Co Ltd database	Inclusion criteria: Type 2 diabetes or unspecified diabetes, prescription of glucose-lowering agent, observation for a continuous period of at least.6 months from January 2005 to July 2014. Mean age 56 years. Exclusions: <18 years or >75 years, type 1 diabetes, severe hypoglycaemia, history of CVD	Oral hypoglycaemic agents, insulin
Haroon et al. (15)	Prospective	Canada, Ontario 225045 with newly diagnosed diabetes 668070 without diabetes	Provincial health administrative databases	Seniors with newly diagnosed diabetes and matched comparison cohort without diabetes aged 66-105 between 1 April 1995 to 31 March 2007. Median age 73 years. Followed until 31 March 2012 for a new diagnosis of dementia Exclusions: dementia at baseline, individuals living in long-term care facilities	Insulin, oral agents
Heller et al. (16)	Post-hoc analysis of EXAMINE trial (RCT)	5380 patients 49 countries	EXAMINE trial	Type 2 diabetes requiring anti-hyperglycaemic medications with a baseline HbA1c of 48-97 mmol/mol or 53-86 mmol/mol if on insulin. Mean age 61 years.	Oral hypoglycaemic agents, insulin
Hsieh et al. (17)	Cohort study	National Health Insurance Database Taiwan 2001–2010 5185 participants–hypoglycaemia group n = 1037; Control group n = 4148	Taiwan National Health Insurance Database Patients with T2DM	Patients aged 20 years and older with T2DM –ICD-9 codes plus three OP visits or more with a diabetes diagnosis code over a 1-year period or at least 1 hospitalization with diagnostic code for T2DM. Patients with one HE constituted the hypoglycaemic group. Excluded: undetermined hypoglycaemia, neonatal/infant hypoglycaemia. Mean age 67.8 years; male 48.4% Control group: patient with T2DM and no HE were matched 4:1 to hypoglycaemic group, based on age and diabetes duration. Mean age 67.8 years; male 52.5%	Insulin and oral hypoglycaemics
Hsu et al. (18)	Prospective cohort	77,611 Taiwan, 1998-2009	Enrolled in National Health Insurance	>60 years, newly diagnosed T2DM (with ≥3 outpatient claims ICD-9-CM code 250). Mean age 65.2 years. Hypoglycaemic patients with randomly selected and matched non- hypoglycaemics.	Insulin, SU, other drugs
Hung et al. (19)	Cohort	Insurance claims database 2001 to 2009 2588 patients Taiwan	National health Insurance Research Database	Type 2 diabetes, mean age 70 years, male 43% Controls were frequency matched on age within 5 years, on gender and on duration of diabetes at a ratio of 1:2 Exclusions: pathological fractures, transportation accident before the index date Median follow-up 3.9 years	Oral hypoglycaemic agents, insulin
Johnston et al. (20)	Retrospective cohort	860,845 US, 30 September 2006 to 30 September 2008	Thomson Reuters MarketScan Commercial Claims and Encounters (Commercial) database and Medicare database	Age>65 years with T2DM (≥1 claim with diagnosis code) and ≥2 prescriptions claims for antidiabetic drugs Continuous enrolment and pharmacy benefits throughout 24-months Exclusion: claim with diagnosis code for T1DM	Oral glucose-lowering agent(s), insulin
Johnston et al. (21)	Retrospective cohort	361,210 1 April 2008 to 31 March 2010	Thomson Reuters MarketScan Commercial Claims and Encounters (Commercial) database and Medicare database	Mean age 75, 52% male, T2DM (≥1 claim with diagnosis code) and ≥2 prescriptions claims for antidiabetic drugs Continuous enrolment and pharmacy benefits throughout 24-month study period.	Any antidiabetic drugs
Kachroo et al. (22)	Retrospective cohort study	43,226 US, 2008-2011	Truven Health Market Scan Medicare Supplemental Database, 21,613 hypoglycaemia patients matched with 21,613 non-hypoglycaemia patients	Type 2 diabetes Randomly matched to controls 1:1 by age, gender Age >65 at index date (first T2DM date in the study period) Male 48%	Metformin, Sulfonylurea, Thiazolodinediones, Insulin
Khunti et al. (23)	Retrospective	Total: 265,868 T2DM: 10,422 England & Wales, 2001-2007	CPRD with hospital episode statistics datalink	All insulin users, age >30 years. T2DM sample mean age 63, male 56% Exclusion: patient without linkage to HES, pre-index period ≤180 days, hypoglycaemia before index, no diabetes classification, CV event at index.	Insulin, with or without other hypoglycaemic drugs
Kim et al. (24)	Cohort study	Korean National Health Insurance Service Senior cohort Korea 2002-2015 48521 participants– 5966 who had experienced hypoglycaemia; 42555 without hypoglycaemia (after propensity-score matching, 5966 patients comprised both groups)	Korean National Health Insurance Service Senior cohort Patients with T2DM	Patients with t2DM and prescribed oral hypoglycaemic agents or insulin. Index date 1 January 2002. Mean age 76 years. Exclusions: T1DM, dementia, dementia medication prescription before the index date (1 January 2002)	Insulin and oral hypoglycaemics
Kong et al. (CKD) (25)	Post-hoc analysis of prospective cohort study	Diabetes Registry 8767 patients Hong Kong	Hong Kong Diabetes Registry (Kong 2014 cancer/mortality)	Type 2 diabetes with and without SH in the 12 months before enrolment Median age 58 years. Exclusions: type 1 diabetes, missing variables used in the analysis	Oral hypoglycaemic agents, insulin
Lee et al. (CV mortality) (26)	Prospective cohort	1209 patients with diabetes who had been recruited for the Artherosclerosis Risk in Communities study (ARIC) (4^th^ study visit in 1996-1998 Is baseline for this analysis) US	ARIC study	Participants with diabetes identified by self-report of a physician diagnosis or use of glucose lowering medication at 4^th^ ARIC study visit. Mean age 64 years. Exclusions: 4 participants who did not identify as black or white and six black participants form the Minnesota or Maryland study site. Excluded those missing covariates. Cause of death was missing for 29 participants, and they were censored at time of death.	No glucose lowering medications, oral hypoglycaemic agents, insulin
Lee et al. (dementia) (27)	Cross-sectional study (cognitive status) ‘Prospective study’-dementia	For the ‘cross-sectional cognitive status’, ‘cross-sectional brain MRI subset’ and ‘prior cognitive decline’ analyses, participants selected from visit 5 (2011–2013) with 2001, 580 and 1755 individuals, respectively. ‘prospective incident dementia’ analysis 1263 participants; baseline at visit 4 (1996–1998), with follow-up to end 2013	ARIC study	Diagnosed diabetes by self-report of diagnosis or diabetes medication use. Mean age 76 years.	No glucose lowering medications, oral hypoglycaemic agents, insulin
Leong et al. (28)	Longitudinal cohort	Primary care Network, 9137 participants Massachusetts, US	Primary care Network	Type 1 or type 2 diabetes patients without coronary artery disease before 1 January 2006; Follow-up until earliest incident of CAD, last clinic visit, death or 30 June 2012. Exposed: 1 or more hypoglycaemic events in 2000-2005; Unexpoosed: no reported hypoglycaemia before 1 January 2006 Mean age 60 years. Exclusions: patients with CAD before 1 Jnauary 2006	Oral hypoglycaemic agents, insulin
Lin et al. (29)	Prospective cohort	Taiwan 15404	National Health Insurance Database	Type 2 diabetes (ICD9-CM) no prior dementia 45% male Mean age 64 [2% had prior hypoglycaemia–not in baseline characteristics]	Oral hypoglycaemics or insulin
Lu et al. (30)	Cohort study Post-hoc analysis of Chen 2010	National Health Insurance Database Taiwan 31049 enrolled in each of three groups	Insurance database	Type 1 and type 2 diabetes plus reference group without diabetes. Diabetes patients divided into those with and those without hypoglycaemia Mean age in patients with diabetes and hypoglycaemia 63 years, 46% male Exclusions: admissions to hospital with cancer or any diagnoses of accident between 1997 to the date of first clinic visit for diabetes in 2000 (=index date) Follow-up; from index date to occurrence of first of date of 1^st^ hospitalization due to falls, date of attrition from insurance or 31 December 2008.	Medications not listed in **Table 1**
Majumdar et al. (31)	Retrospective cohort	85,810 Canada, 2004-2009	Alberta Kidney Disease Network and the provincial health ministry (Alberta Health)	Outpatients age>66 years (mean 75) who had administrative data for both serum creatinine and HbA1c within 6 months of each other 51% female 50% diabetic	Oral hypoglycaemic drugs (mono therapy or combination), insulin
Mattishent et al. (3)	Retrospective cohort	Primary care database 1997-2016 19993 patients	CPRD database	Patients aged 65 or older with diabetes, defined as a first ever prescription of any oral or injectable glucose-lowering agent between April 1997 and March 2016. Follow-up continued for up to five years from the exposure, loss from database, death, or end of available database linkage (HES 31 March 2016 and ONS 17 April 2017), whichever was the earlier	Insulin, oral hypoglycaemic drugs
McCoy et al. (32)	Retrospective cohort	1013 Diabetes Clinic, single centre, US August 2005–July 2006	Medical records	Type 1 and Type 2 diabetes, mean age 60.5 years, male 55%, history of hypoglycaemia established prior to index clinical encounter Exclusions: seven lost to follow-up	Insulin, oral hypoglycaemic drugs
Mehta et al. (33)	Retrospective cohort	Primary care database 53055 patients 2003-2012 UK	CPRD	New diagnosis of type 2 diabetes from 2003-2012, >65 years Exclusions: patients with dementia diagnosis in a year prior to index date (=1^st^ date of diabetes diagnosis or a laboratory value indicating diabetes diagnosis), patients with no information on baseline HbA1c and untreated patients	Oral hypoglycaemic agents, insulin
Mellbin et al. (34)	Posthoc analysis of RCT	12,537 40 countries, 2003-2005	International multicenter randomized controlled trial of two different interventions in dysglycemic individuals with IFG, IGT, newly detected diabetes, or established diabetes	50 years or older (mean age 63.5, 65% male) with cardiovascular risk factors 60% had prior cardiovascular event, 80% had prior diagnosis of diabetes, 6% had newly detected Type2 diabetes, 12% had impaired glucose tolerance or impaired fasting glucose. Median baseline HbA1c 6.4% and fasting plasma glucose 6.9mmol/L	Insulin glargine, oral hypoglycaemic drugs
Ntouva et al. (35)	Retrospective cohort	Primary care database 1995-2016 41163 participants UK	The Health Improvement Network (THIN)	Type 2 diabetes aged 18 and older, registered in general practices contributing to THIN between 1 January 1995 to 1 May 2016 Mean age 67 years. Follow-up: earliest of transfer date, death date, first documentation of outcome (fracture) or study end date) History of hypoglycaemia = exposed cohort No history of hypoglycaemia = unexposed cohort. Exclusions: history of fracture	Oral hypoglycaemic agents, insulin
Pieber et al. (36)	Post hoc analysis of RCT	DEVOTE RCT–multicentre, prospective, treat-to-target,double-blind, active comparator cardiovascular outcomes trial 7637 patients randomised to either insulin degludec or insulin glargine	DEVOTE trial	Type 2 diabetes with at least one oral or injectable glucose-lowering agent with HbA1c >7.0% (53mmol/L) or with >20 units/day basal insulin. Either had at least one co-existing cardiovascular or renal condition and were aged >50 years or had at least one of a list of pre-specified cardiovascular risk factors and were aged >60 years. NOT excluded if experienced SH prior to randomisation. Mean age 65 years.	Oral hypoglycaemic agents, insulin
Rajpathak et al. (37)	Retrospective cohort	42,747 US, (1 January 2002 to 31 December 2005), with 13195 propensity matched pairs	OptumInsight, medical claims database	>65 years (mean 72.5 years) with T2DM 1:1 propensity matching score (Sulfonylurea v non-sulfonylurea users) Exclusion criteria: drug supply <30 days, insulin use, prior hip fracture, SU initiation among non-users or discontinuation among users after the index date	SU
Rathmann et al. (38)	Retrospective	19184 DPP-4 and 31110 SU users (total: 50294) Germany (1201 general practices), April 2007 to July 2010	Primary care data: Disease Analyzer Database (IMS HEALTH)	Type 2 diabetes with first time prescription (index date) of either DPP-4 inhibitors or SU from Continuous treatment in same practice Mean age 64 (DPP-4) and 69 (SU) Excluded: use of both SU and DPP-4 inhibitor; insulin use at baseline or follow-up, or any other antidiabetic drugs except metformin	DPP-4 SU
Signorovitch et al. (39)	Retrospective	33,492 US, 1998-2010	Claims database from self-insured companies	T2DM who had filled ≥2 prescriptions for oral hypoglycemic drugs Mean age 60; Male 50% Random sample without hypoglycaemia 5:1 ratio to hypoglycaemic patients Exclusion: evidence of insulin use	Oral hypoglycaemic drugs
Standl et al. (40)	Post-hoc analysis of RCT	TECOS trial (tiral evaluating cardiovascular outcomes with sitagliptin) 14671 participants; multi-national, double-blind, placebo-controlled, randomized, event-driven trial designed to assess CV safety of sitagliptin vs placebo	TECOS trial	Type 2 diabetes, pre-existing coronary, cardiovascular or peripheral atherosclerotic disease, >50 years, baseline HBA1c 6.5-8% (48-64mmol/L) Mean age 65 years. Exclusion: those on DPP4 inhibitor, GLP-1 agonist, Rosiglitzone during the preceding three months, >2SH episodes in the previous 12 months, eGFR <30 ml/min Follow-up median of 3 years	Oral hypoglycaemic agents, insulin
Whitmer et al. (41)	Prospective	US 16667	Kaiser Permanente Northern California Diabetes Registry (1980–2007)	Type 2 diabetes Mean age 66 Male 55% No prior diagnoses of dementia, mild cognitive impairment, or general memory complaints.	Insulin, oral agents
Yaffe et al. (42)	Prospective	US 783	Participants with DM enrolled in Health, Aging, and Body Composition Study. Excluded those with evidence of possible cognitive impairment at study baseline	DM (self-report, use of hypoglycaemia meds, or biochemical testing) Mean age 74 47% black ethnicity 52.4% male Baseline modified MMSE >80 (no pre-existing cognitive impairment)	Insulin, oral agents
Zhao et al. (43)	Retrospective cohort study	44,261 (unmatched sample), 761 hypoglycaemia matched to 761 controls US, January 2004- September 2010	Electronic medical and pharmacy records Veteran Health Administration	Type 2 diabetes, mean age 63, male 96% Excluded: patients with 1-year pre-index records of hypoglycaemia, cardiovascular, and microvascular diseases, patients with T1DM	Oral glucose lowering agents, insulin
Zhao et al. (44)	Retrospective cohort study	Cohort 4215 with hypoglycaemia matched to controls US January 2004-July 2010	Electronic medical and pharmacy records Veteran Health Administration	Mean age 76.5, Type 2 diabetes Excluded: patients with T1DM, patients with 6-month pre-index record of fall	Oral glucose lowering agents, insulin
Zinman et al. (45)	Post-hoc analysis of RCT	9430 patients Multi-centre, double-blind, placebo-controlled RCT	LEADER trial	Type 2 diabetes; age 50 years or older and established CV disease or chronic renal failure OR age >60 years and risk factors for CV disease, HbA1c >7% Randomized to liraglutide or placebo both in addition to standard-of-care. Follow-up 3.5-5 years. Mean age 65 years. Exclusions: type 1 diabetes, use of GLP-1 agonist, DDP-4 inhibitors, pramlitinide, or rapid-acting insulin, history of MEN type 2 or medullary thyroid cancer and occurrence of an acute coronary or cerebrovascular event.	Oral hypoglycaemic agents, insulin
Zoungas et al. (46)	Posthoc analysis of RCT	11,140, 215 centres in 20 countries June 2001 to March 2003	ADVANCE randomized controlled trial of intensive glucose lowering (between)	Type 2 diabetes, ≥55 yrs, diagnosis after the age of 30 and had a history of macrovascular or microvascular disease or at least one other cardiovascular risk factor. Mean age 66 years. Excluded if clear indication for long-term insulin at baseline.	Gliclazide together with other oral glucose lowering agent(s)

OR, Odds ratio; HR, Hazard ratio; 95% CI, 95% Confidence Interval; T2DM,Type 2 diabetes mellitus; HbA1C,glycated haemoglobin; CKD, chronic kidney disease; DPP-4,dipetidyl-peptidase-4; SU, Sulfonylureas; MI, myocardial infarction; AF, atrial fibrillation; CVD, cardiovascular disease; HES, Hospital Episode Statistic; CPRD, Clinical Practice Research Datalink database; TUG, Timed Up and Go Test; GDS-15,Geriatric Depression Scale; CCI, Charlson comorbidity index; IFG, impaired fasting glucose; IGT, impaired glucose tolerance; SH, severe hypoglycaemia; ACEi, angiotension-converting enzyme inhibitor; ARB, angiotension receptor blocker; TIA, transient ischemic attack; MMSE, Mini-Mental State Examination; eGFR, estimated glomerular filtration rate.

**Table 2:** Study outcomes, results and risk of bias.

Study ID	Method of diagnosing each type of adverse event	Method of diagnosing or determining that patients had hypoglycaemia	Statistical adjustments for confounding factors (if any)	Results
Bedenis et al. (4)	Primary outcomes were MI (fatal or nonfatal), angina, TIA, stroke (fatal or nonfatal). Composite macrovascular disease outcome defined as one or more episodes of MI, angina, TIA or stroke. Coronary heart disease defined by occurrence of MI or angina. Cerebrovascular disease defined by occurrence of stroke or TIA. Self-report questionnaire or *via* GP questionnaire, WHO chest pain questionnaire, ECG and hospital discharge data linkage from Information and Services Division of NHS Scotland	Self-report questionnaire (severe hypoglycaemia only)	Univariate and multivariate-adjusted regression models adjusting for age, sex, blood pressure, HbA1c, total cholesterol, HDL cholesterol, BMI, eGFR, duration of diabetes, smoking status, diabetes treatment method, medications, microalbuminuria	Macrovascular disease events aOR 2.11 (1.06–4.21); Coronary heart disease events aOR 2.44 (1.13–5.26); Cerebrovascular disease events aOR 2.01 (.029–3.61); MI aOR 4.02 (1.54–10.48); Stroke aOR 0.86 (0.21–3.56)
Bonds et al. (5)	Pre-specified primary outcome: non-fatal MI or non-fatal stroke and cardiovascular death Pre-specified secondary outcome: all cause mortality Blinded independent adjudication of outcomes	Investigators asked patients about hypoglycaemic events at each visit. Patients were given home glucose monitors: -symptomatic severe hypoglycaemic event requiring medical assistance (HMA); blood glucose <2.8mmol/L or symptoms resolved with treatment -symptomatic severe hypoglycaemic event requiring any assistance (HA)	Cox regression models (stepwise procedure) Confounders: baseline covariates, age, gender, ethnicity, education, BMI, alcohol, smoking, cardiovascular disease, diabetes duration, diabetic complications, cardiovascular risk factors, medication, trial treatment assignment	Association between any hypoglycaemic event and mortality intensive arm aHR 1.41 (1.03, 1.93) standard care arm aHR 2.30 (1.46, 3.65)
Cha et al. (6)	Primary outcome: death from any cause or cardiovascular death (deaths resulting from acute MI, sudden cardiac death, death due to heart failure, other CV causes) CVD based on review of medical records and diagnosis confirmed by cardiologist, neurologist or neurosurgeon. Causes of death determined from death certificates, clinical records and hospital records	SH–hypoglycaemic episodes requiring the assistance of medical care in an emergency department or hospitalization. Patients were asked if they had experienced SH; review of medical records	Cox proportional hazards regression with adjustments for: sex, age, duration of diabetes, hypertension, diabetic nephropathy, mean HbA1c, unsulin, ACEi, ARB, CVD history	Cardiovascular mortality aHR 6.34 (2.02–19.87) All-cause mortality aHR 2.64 (1.39–5.02)
Chiba et al. (7)	Professional interviewer with questionnaire about frequency and type of falls (defined as unexpected event in which the person came to rest on the ground, floor, lower level. Complicated with a head injury or fractures).	Professional interviewer with validated questionnaire regarding hypoglycaemic symptoms. Severe: coma, convulsion, inability of self-management and recovery from symptoms. Mild: hypoglycaemic symptoms with recovery within 10 minutes by self-administered sugar or glucose.	Multiple regression analysis: age, sex, cognitive impairment (MMSE <26), TUG score, GDS-15 scores, Falls Risk Index, presence of hypoglycaemia.	Presence of hypoglycaemia OR 3.62 (1.24, 10.53), associated with presence of multiple falls, and any fall OR 2.05 (0.93–4.535). Prevalence of falls increased as the frequency of hypoglycaemia increased.
Chin et al. (8)	Incident cases of dementia and organic mental disorder were identified form HIRAS claim database (ICD-10 codes)	HIRAS claims database (ICD-10 codes)	Cox proportional hazards regression models adjusting for age, sex, smoking status, alcohol status, BMI, diastolic blood pressure, medications, diabetes duration, dyslipidaemia, CVD, cerebrovascular disease	Any events of hypoglycaemia and risk of dementia aHR 2.689 (1.080–6.694) Two or more hypoglycaemic events and risk of dementia aHR 4.065 (1.099–15.039)
Cukierman-Yaffe et al. (9)	Incident cognitive dysfunction defined either as reported dementia (first occurrence of an affirmative answer to a case report form question) or a post-randomization MMSE score of <24. Sensitivity analysis conducted using a more restrictive definition of cognitive dysfunction (reported dementia or two consecutive MMSE scores <24 or last available MMSE score <24)	Self-reported questionnaires. Nonsevere hypoglycaemia defined as an event associated with symptoms consistent with hypoglycaemia and confirmed by a capillary glucose reading of <54mg/dL (3mmol/L). SH defined as a symptomatic events requiring assistance of another person and there was prompt recovery after oral carbohydrate, IV glucose or glucagon and.or documented self-measure or laboratory-measured plasma glucose level of <36 mg/dL (2 mmol/L)	Cox proportional hazards regression adjusting for baseline CVD, diabetes status, allocation to glargine, allocation to b-3 fatty acids, HbA1c as a time-varying covarite, age. Accounted for competing risk of death.	Relationship between SH and incident cognitive impairment after adjusting for baseline CVD, diabetes status, treatment allocation: aHR 1.16 (0.89–1.52) Model with propensity score for SH: aHR 1.00 (0.76–1.31) Sensitivity analysis with more restrictive definition of cognitive impairment: aHR 1.21 (0.90–1.63) Non-SH and risk of incident cognitive impairment aHR 0.59 (0.52– 0.68); Model with propensity score for non-SH aHR 0.58 (0.51–0.67). Sensitivity analysis with more restrictive definition of cognitive impairment aHR 0.62 (0.52–.073)
Davis et al. (10)	Primary outcome: time to first occurrence of any one of a composite of cardiovascular events adjudicated by an end point committee that was unaware of study group assignments. Cardiovascular events were documented MI, stroke, death as a result of cardiovascular causes, new or worsening congestive cardiac failure, surgical intervention for cardiac, cerebrovascular, or peripheral vascular disease, inoperable coronary artery disease and amputation for ischaemic gangrene. Total mortality pre-specified secondary outcome.	SH defined as a self-reported episode of a low blood glucose value accompanied by confusion requiring assistance from another person of loss of consciousness.	Cox proportional hazards regression adjusting for treatment group, overall cardiovascular risk (including factors such as diabetes duration, HbA1c), prior cardiovascular event, insulin use, eGFR	SH within prior three months and association with cardiovascular events and mortality Cardiovascular events aHR 1.90 (1.06–3.52) Cardiovascular mortality aHR 3.7 (1.30–10.40) All-cause mortality aHR 2.40 (1.10 to 5.10)
Duckworth et al. (11)	Cardiovascular event is pre-specified composite: MI, stroke, CV death, cardiac failure, vascular surgery, inoperable coronary artery disease, amputation for gangrene Blinded independent adjudication of outcomes	Routine trial monitoring	Multivariate regression analysis Confounders: prior cardiovascular event, age, baseline insulin, ethnicity, smoking status, HbA1c, lipids, creatinine, diabetes treatment and duration	HR for composite cardiovascular event 1.88 (1.029, 3.432)
Escalada et al. (12)	Outcomes: medically attended hypoglycaemia events Hospitalization Secondary outcome: mortality	Medically attended hypoglycaemia events identified from claims database–ICD-9 codes	Cox proportional hazards regression for risk of hospitalization with medically attended hypoglycaemia as the time-varying covariate, adjusting for demographic, comorbidity and medication history factors Three sensitivity analyses for mortality modelling after hypoglycaemia: 1) mortality risk amongst the population with an MI, congestive heart failure, peripheral vascular disease or stroke; 2) population with MI, CHF, PVD, stroke, dementia or renal disease,3) population without cancer	Medically attended hypoglycaemia after initiation of basal insulin and risk of hospitalization aHR 1.59 (1.53–1.65) Hypoglycaemia and risk of death aHR 1.50 (1.40–1.60) Sensitivity analyses 1) aHR 1.46 (1.34–1.58) 2) aHR 1.44 (1.34–1.56) 3) 1.48 (1.37–1.58)
Freemantle et al. (13)	Primary outcomes: composite of stroke of myocardial infarction or cardiovascular-specific death; relationship between reported hypoglycaemic episodes and CV or all-cause mortality Cardiovascular events reported by investigator in clinical report forms at 6-month intervals; supportive documents requested by study team (ECG, hospital records, biochemistry, radiology reports, medication charts) Cardiovascular events: MI, stable angina, severe unstable angina leading to hospitalization, stroke, TIA, PVD, limb amputation, myocardial revascularization, peripheral revascularization	Reported by participants Data were gathered in routine clinical practice, and treating physicians were asked to report updated participant data every 6 months	Cox proportional hazards regression Time-to-event endpoints calculated from date of insulin initiation and were restricted to 54 months	Relationship between reported hypoglycaemia and CV death or all-cause mortality CV death and SH aHR 1.10 (0.34–3.57) All-cause mortality and SH 1.22 (0.59–2.53)
Goto et al. (14)	Primary outcomes: severe hypoglycaemia and CVD CVD defined as conditions during hospitalization with both a diagnosis of CVD (ischaemic heart disease, stroke, peripheral artery disease) and either a medical procedure performed or a prescription to treat CVD	Severe hypoglycaemia defined by ICD 10 code and prescription for either 50% dextrose or glucagon infusion	Cox proportional hazards models to evaluate association of SH with CVD risk, adjusted for age, sex, duration of diabetes, history of microvascular disease, Charlson Co-morbidity index, medications. 5:1 propensity score matching	Association between SH and CVD risk aHR 3.39 (1.25–9.18) Propensity-score matched cohort: aHR 7.31 (1.87–28.6)
Haroon et al. (15)	Dementia: defined based on one or more hospitalisation records or two outpatient physician billing claims (within six months) listed relevant ICD-9 claim	Healthcare administrative database records of hospitalisation or emergency department visits for hypoglycaemia	Cox proportional hazard modelling Sensitivity Analyses to examine whether detection bias could explain elevated risk of dementia Models were adjusted for baseline income and co-morbidities, including hypertension, chronic kidney disease and vascular diseases of varying aetiologies. Cumulative incidence functions were used to estimate the probability of occurrence of dementia	Hospitalisation and emergency department visits for hypoglycaemia were significant predictors of dementia aHR 1.73 (1.62–1.84) based on comparison of one or more episodes versus none.
Heller et al. (16)	Primary endpoint in EXAMINE trial: composite of death from cardiovascular causes, nonfatal MI or non-fatal stroke. Principal secondary safety endpoint was primary composite end point with addition of urgent revascularization due to unstable angina within 24 hours after hospital admission. Exploratory endpoints included death from cardiovascular causes and death from any cause. Independent central adjudication committee adjudicated all suspected primary end-point events and other cardiovascular and points, as well as all deaths.	Assessed at study visits at 1, 3, 6, 9 and 12 months post-randomization during the first year of the study and every 4 months during subsequent years of participation. Hypoglycaemic events characterized by local investigators according to their intensity (mild to severe) and seriousness (hospitalization or ED management)	Cox proportional hazards models with adjustments for age, sex, treatment, HbA1c, glycaemic medication and stratified by screening renal function and geographic region	Risk of MACE based on reported serious hypoglycaemia aHR 1.60 (0.80–3.20)
Hsieh et al. (17)	Primary endpoint: ventricular arrhythmia requiring hospital visit, including VT, VF or sudden cardiac arrest (SCA)–all ICD-9 codes	ICD-9 codes	Cox Proportional Hazard Regression Models with adjustments for age, gender, AF, sinus node disease, premature ventricular contractions, stroke HTN, hyperlipidaemia, coronary artery disease, COPD, heart failure, VHD, PVD, CKD, ACEi, ARB, beta-blockers, aspirin, clopidogrel, warfarin, statins, digoxin, diabetes medications	aHR for any hypoglycaemia = 2.32 (0.97–5.53)
Hsu et al. (18)	Cancer, stroke, coronary heart disease and cardiovascular disease identified from hospital claims dataset, ICD-9-CM codes Death status ascertained according to discharge reasons with death or critically ill at discharge, or if insurance cover stopped due to death.	Hospital claims dataset for severe hypoglycaemia Outpatient claims dataset for mild hypoglycaemia ICD-9-CM codes	Propensity score, Cox proportional hazard model, Kaplan-Meier Variables in propensity score matching: age, sex, diabetes duration, hypertension, heart disease, renal and liver disease, cancer, mental disease, socio-economic status, treatment adherence.	HR 2.09 (1.63, 2.67) for cardiovascular diseases, HR 2.51 (2.00, 3.16) for all-cause hospitalisation, HR 2.48 (1.41, 4.38) for total mortality
Hung et al. (19)	Primary outcome: hip fracture after SH Insurance claims ICD-9	Index date for SH was first date of the development of hospitalized or ED hypoglycaemic visit Insurance claims ICD-9	Cox proportional hazards regression models adjusted for sex, ESRD, COPD, epilepsy, CAD, stroke, dementia, Parkinsons, osteoporosis, retinopathy, neuropathy, alcohol misuse, TZD, estrogen, acarbose, glinide, metformin, SU, DPP4i, beta blocker, corticosteroid, anti-depressants, NSAIDs, anti-osteoporosis	Risk of hip fracture higher in relation to SH aHR 1.71 (1.35–2.16)
Johnston et al. (20)	Acute cardiovascular events: coronary artery bypass graft, revascularisation, percutaneous coronary intervention –≥one inpatient or outpatient claim ICD-9-CM code Acute MI, incident unstable angina–≥1 inpatient claim with an ICD-9-CM code	≥1 outpatient claim with ICD-9-CM diagnosis code for hypoglycaemia (hypoglycaemic events were allowed to occur at any time during the evaluation period, including after acute cardiovascular events)	Multiple logistic regression and backwards stepwise selection Adjusted for age, sex, geography, insurance type, comorbidity scores, cardiovascular risk and prior events, diabetes complications, total baseline medical expenditures.	OR 1.79 (1.69, 1.89) for acute cardiovascular events Patients >age 65 years OR1.78 (1.65, 1.92)
Johnston et al. (21)	Emergency department claim with ICD-9-CM diagnosis code	≥1 outpatient claim with ICD-9-CM diagnosis code for hypoglycaemia (hypoglycaemic events allowed to occur at any time during evaluation period, including after fracture)	Multiple logistic regression Confounders: patient demographics, baseline co-morbid conditions, baseline medications, CCI, medical encounters for diabetes, total baseline medical expenditures, number of medical codes	aOR for fall-related fractures 1.70 (1.58, 1.83)
Kachroo et al. (22)	Admin claim data Fall-related events defined as ICD-9-CM codes 800.x-995.x, with a fall being the external cause defined as ICD-9-CM E-codes E880-E888 which were recorded within +/-2 days of each other in any order. Composite fall events (e.g. fall with head injury or fracture) identified based on two or more claims codes occurring within 2 days.	Admin claim data ICD-9-CM codes 250.8, 251.0, 251.1 and 251.2	Logistic regression analysis Patients matched on age and gender; statistical adjustment on CCI	Risk of fall-related events aOR 1.95 (1.70, 2.2) Fracture–aOR 2.16 (1.74–2.67)
Khunti et al. (23)	Cardiovascular event defined as a composite of MI, stroke or cardiovascular death (cause of death obtained through linkage to Office for National Statistics).	Data on hypoglycaemic episodes were obtained from HES *via* ICD-10 codes 9E16.0, E16.2)	Mutivariate Cox regression models Covariates: age, sex, smoking status, geographical region, history of cardiovascular events before index date, use of oral antidiabetic medications, Charlson comorbidity index, BMI, HbA1c	All-cause mortality for T2DM: HR 1.94 (1.52, 2.47) and 2.39 (2.13, 2.67) for those with and without a history of CVD Cardiovascular events for T2DM: HR 1.70 (1.09, 2.64) and 1.50 (1.19, 1.88) for those with and without a history of CVD
Kim et al. (24)	Primary outcome: first diagnosis of all-cause dementia–ICD-10 codes. Secondary outcome: first diagnosis of Alzheimer’s dementia or Vascular dementia– ICD-10 codes.	ICD-10 codes	Propensity-Score matched analysis; confounding variables set as: age, gender, socioeconomic status (at index date), diagnoses (1 yr before index date) (hypertension, dyslipidaemia, end-stage renall disease, malignancy, asthma, COPD, connective tissue disease, AF, heart failure, osteoporosis, cerebrovascular disease, microvascular complications of diabetes), medication use, obesity, alcohol use.	Risk of all-cause dementia for any hypoglycaemia aHR 1.25 (1.17–1.35)
Kong et al. (CKD) (25)	All clinical outcomes ascertained though Hospital Authority Central Computer Management System, which records diagnoses of all hospital discharges, including morality based on ICD-9 codes. Mortality data cross-checked with Hong Kong Death Registry. Cause of death was defined by the principal discharge diagnosis.	SH defined as one or more hospitalizations for hypoglycaemia in the 12 months before enrolment or during the follow-up period from enrolment to death or 31 January 2009	Cox proportional hazards regression models with adjustments for age, sex, BMI, smoking satus, alcohol use, LDL-C, HDL-C, systolic BP, HbA1c, duration of diabetes, urinary albumin to creatinine ratio, prior CVD, prior cancer, medications at enrolment.	Hazard ratios of severe hypoglycaemia for the risk of all-cause death in patients with type 2 diabetes aHR 1.81 (1.38–2.37)
Lee et al. (CV mortality) (26)	An expert committee adjudicated all coronary heart disease and stroke events. Adjudication for heart failure began in 2005; all heart failure events before 2005 were based on hospitalization with a primary position ICD-9 code. Incident atrial fibrillation was based on hospitalizations with ICD-9 codes for atrial fibrillation or atrial flutter. Mortality was assessed *via* proxy, coroner reports, and the National Death Index through 2013. Cause-specific mortality was classified by the underlying cause of death listed on the death certificate.	Severe hypoglycemic events were identified from hospitalizations, emergency department visits, and ambulance calls with a validated algorithm, using ICD-9 codes in the primary position through 31 December 2013. Hospitalization records were available from ARIC surveillance of local hospitals. Linked Medicare claims for hospitalizations, emergency department visits, and ambulance use were available for participants enrolled in Medicare fee-for-service part B.	Cox proportional hazards regression models adjusting for age, sex race-centre, diabetes medication use, duration of diabetes, tertiles of fructosamine, low eGFR, albumin-urea ratio, income, disability, systolic BP, hypertension, LDL-C, HDL-C, medications, smoking status (Model 3)	Association between SH and CV events and all-cause mortality (Model 3) Coronary heart disease aHR 2.02 (1.27–3.20) Stroke aHR 0.81 (0.40–1.63) All-cause mortality aHR 1.73 (1.38–2.17)
Lee et al. (dementia) (27)	Assessment of cognitive status (normal, mild cognitive impairment or dementia) was based on available cognitive test scores from visits 2 (1990–1992), 4 (1996–1998) and 5 (2011–2013), the Clinical Dementia Rating (CDR), based on interviews with participants and informants, the Modified Telephone Interview for Cognitive Status (TICS), hospitalisation records and death certificates. Diagnoses were standardised using an algorithm, with review by a panel of experts, who overrode the algorithm if indicated by their clinical judgement. For the analysis of incident dementia, a date of dementia diagnosis was assigned as the date of hospitalisation with a dementia ICD-9 code or, if no hospitalisation with dementia occurred, the first date of detection via the TICS or CDR, or visit 5.	Severe hypoglycaemic episodes were identified from hospitalisations, emergency department visits and ambulance calls by a widely used algorithm that employs primary position ICD-9 codes	Multinomial logistic regression to compare the odds of having mild cognitive impairment or dementia by history of severe hypoglycaemia For the prospective incident dementia analysis, we used a Cox regression model for the outcome of incident dementia, with severe hypoglycaemia as a time-varying exposure (4 models used adjusting for covariates)	Prospective association of severe hypoglycaemia with incident dementia among ARIC participants with diagnosed diabetes at visit 4 in the prospective incident dementia analysis Model 4 aHR 2.28 (1.58–3.29)
Leong et al. (28)	Electronic health record repository, including outpatients, emergency department and inpatient visits. iCD-9 code-cased algorithm	Electronic health record repository, including outpatients, emergency department and inpatient visits. Hypoglycaemia defined as hypoglycaemia brought to medical attention. ICD-9 code-based algorithm	Three Cox models for incident CAD constructed, Model 3 fully adjusted adjusting for sex, age, educational attainment, CAD risk factors, insulin, oral hypoglycaemics, total medication count, retinopathy, neuropathy, renal failure, eGFR, LDL < HDL, cancer, dementia, dysrhythmias, hospitalizations, weight loss within a year, HbA1c measurements per year.	Hypoglcyaemia and associated with CAD risk aHR 1.90 (1.09–3.31) Risk after adjusting for socio-demographic characteristics, hypertension, dyslipidaemia, diabetes duration, BMI and HbA1c aHR 2.15 (1.24–3.74) Fully adjusted model aHR 1.65 (0.95–2.87)
Lin et al. (29)	Dementia: ICD9-CM Method of diagnosing not stated	ICD9-CM Method of diagnosing not stated	Multivariable Cox proportional hazard analysis Age, gender, co-morbidities (Ischaemic heart disease, cardiovascular disease, hyperlipidaemia, chronic renal disease, hypertension), insulin use.	Adult diabetic patients with prior hypoglycaemia had a significantly increased risk dementia: aHR 1.45 (1.07–1.97);
Lu et al. (30)	Falls needing admission to hospital–fall-related diagnosis code in discharge diagnosis during the follow-up (ICD-9 codes). Unable to distinguish between the falls occurring before or during hospitalization.	SH defined as presence of ICD-9 codes in outpatient and inpatient visits before the index date	Proportional hazards regression models Large number of deaths during follow-up: mortality as competing risk Fine & Gray competing risks model Sequential construction of multivariate regression. Adjustments for age, sex, type of diabetes, geographic area, urbanization status, obesity, mental health problems, neurological, cardiovascular, endocrine, renal, ophthalmic disorders epilepsy, stroke, substance abuse	Risk of falls in diabetes with hypoglycaemia group (patients without diabetes as referent group) >65 years aHR 1.33 (1.21–1.47) Patients without hypoglycaemia as referent >65 years aHR 1.35 (1.25–1.45)
Majumdar et al. (31)	Primary outcome: all-cause mortality Secondary end points included all-cause hospitalisations and hypoglycaemia-associated hospitalisations. Mortality and dates of hospitalisation determined by linkage to provincial health ministry databases.	Defined severe hypoglycaemia by the presence of any inpatient discharge diagnosis of hypoglycaemia (ICD-10 code E15 or E16)	Multivariable Cox proportional hazard methods Adjusted for age, sex, socioeconomic status (based on individual health insurance premium level and median neighbourhood income), index eGFR, prevalent hypoglycaemia, co-morbidities, use of diabetes medications	Mortality associated with any hospitalisation with hypoglycaemia in patients with diabetes: aHR 2.46 (2.17, 2.80)
Mattishent et al. (3)	Outcomes were falls, fractures, cardiovascular events (myocardial infarction, ischemic stroke) and all-cause mortality. Data obtained from CPRD using Read codes and HES with ICD codes	First hypoglycaemic episode recorded on the primary (CPRD) or secondary (HES) healthcare database from April 1997 onwards following initiation of a glucose-lowering agent. Data on hypoglycaemic episodes were obtained from CPRD using Read codes and HES with ICD codes	Cox proportional hazard regression models with adjustments for medications, age, gender, co-morbidities, Townsend deprivation index	Hypoglycaemia was associated with an increased risk during 12 months follow-up of: Falls 1.96 (1.69–2.29) Fractures 1.62 (1.25–2.08) Cardiovascular events - aHR 2.00 (95% CI 1.61 to 2.48) Mortality - aHR 2.36 (95% CI 2.09–2.67)
McCoy et al. (32)	Ascertainment of mortality from medical records and social security death index	Investigator asked patients about hypoglycaemic events -mild hypoglycaemia: symptoms consistent with hypoglycaemia not requiring any assistance -severe hypoglycaemia: similar symptoms requiring external assistance	Logistic regression Confounders: age, gender, type of diabetes and duration, CCI, HbA1c	OR 3.381 (1.547, 7.388) Association between severe hypoglycaemia and 5-year mortality
Mehta et al. (33)	Outcome variable was time to dementia–defined by diagnosis codes from electronic medical records.	Hypoglycaemia defined based on previously defined algorithm for CPRD data. Modelled as time-dependent variable. Read and Med codes	Fine and Gray’s competing risk model–extension of Cox model to competing risk data by considering subdistribution hazards. Adjustments for: age, sex, HbA1c, alcohol use, smoking status, diabetes treatment, co-morbidities associated with dementia. Follow-up from 1^st^ date of diabetes diagnosis until they developed dementia–censored at earliest of: death, loss from database or at 31 December 2012	Association of hypoglycaemia with dementia Fully adjusted model aHR 1.27 (1.06–1.51)
Mellbin et al. (34)	-Composite of cardiovascular death (any death for which no non-cardiovascular cause could be identified), non-fatal MI (based on clinical presentation, elevated cardiac markers, and/or new electrocardiographic changes), or stroke (based on clinical presentation and imaging) -Mortality Blinded independent adjudication of outcomes	Participants recorded hypoglycaemic events with glucose meters and diaries. Investigators asked patients about hypoglycaemic events at each study visit. Non-severe hypoglycaemia: relevant symptoms confirmed by glucose reading <3mmol/L. -severe hypoglycaemia: symptomatic hypoglycaemia requiring assistance of another person with (i) prompt recovery after oral carbohydrate and/or (ii) documented plasma glucose level <2mmol/L	Propensity score matching, as well as Cox regression models addressing potential confounders: age, gender, ethnicity, education, prior cardiovascular events, hypertension, depression, current smoking, alcohol intake, albumin/creatinine ratio >30 mg/g, diabetes and cardiovascular drugs, BMI, waist-hip ratio, HbA1c, fasting plasma glucose, lipids, serum creatinine, mini-mental status, prior diabetes mellitus	In those with severe hypoglycaemia HR 1.58 (1.24, 2.02) for composite event. HR 1.71 (1.27, 2.30) for cardiovascular death. HR 1.74 (1.39, 2.19) for total mortality.
Ntouva et al. (35)	Primary outcome: any fracture; secondary outcome: fragility fracture Read codes obtained from database	Read codes obtained from database	Incidence of outcome of interest was compared between exposed and unexposed group. Incidence Rate Ratios derived using Poisson regression adjusting for covariates: age, sex, BMI, Townsend deprivation index, smoking, CCI, HbA1c, insulin, bisphosphonates, steroid, hyperthyroidism, Graves disease, renal impariement, antihypertensive medications.	Risk of all fractures in patients with documented hypoglycaemia compared to those without aIRR 1.20 (1.12–1.30)
Pieber et al. (36)	Primary outcome: MACE (cardiovascular death, non-fatal MI, non-fatal stroke)	Adjudication-confirmed SH was pre-specified, multiplicity-adjusted secondary outcome as defined by ADA as an episode requiring the assistance of another person to actively administer carbohydrates or glucagon, or to take other corrective action.	Cox regression models Adjustments for age, sex, HbA1c, BMI, diabetes duration, insulin, hepatic impairment, renal status, cardiovascular risk group	Risk of MACE for individuals who had vs those who had not experienced SH aHR 1.38 (0.96–1.96) All-cause mortality aHR 2.51 (1.79 to 3.50)
Rajpathak et al. (37)	Hip fracture defined as an ICD-9 code 820.xx	ICD-9 codes based on validated algorithm	Multivariable logistic regression based on propensity score as well as adjustment for confounders:: age, sex, Medicare cover, region, coronary heart disease, stroke, osteoporosis, dementia, CKD	aOR 2.42 (1.35, 4.34) for hip fractures in those with documented hypoglycaemia
Rathmann et al. (38)	Macrovascular complications were determined based on primary care diagnoses (ICD-10 codes) for coronary heart disease (I20, I24 and I25), MI (I21, I22, I23 and I25.2), stroke (I63, I64, G45) and peripheral vascular disease (E10.5, E11.5, E14.5 and I73.9)	ICD-10 codings (E16.0, E16.1, E16.2) Frequency of patients with >1 hypoglycaemic event assessed 30, 90, 183, 365 and 730 days after index date	Adjusted for age, sex, type of practise (diabetologist), practise region, health insurance status (private), antidiabetic co-medication, episodes of hypoglycaemia, microvascular complications, hypertension, hyperlipidaemia, antihypertensive, lipid-lowering and antithrombotic drugs and Charlson co-morbidity index	HR 1.6 (1.1, 2.2) for incident macrovascular complications
Signorovitch et al. (39)	Inpatient and emergency department claims based on ICD9-CM codes, grouped into three codes: accidental falls, motor vehicle accidents and other accidents	ICD-9-CM codes for hypoglycaemia at any place of service	Multivariable Cox-proportional hazard models adjusted for age, gender, demographics, co-morbidities of diabetes, accident risk factors, CCI, inpatient admissions, use of oral hypoglycaemics.	Hypoglycaemia associated with accidental falls aHR 1.36 (1.13, 1.65) For age >65: aHR 1.52 (1.18, 195)
Standl et al. (40)	Primary 4-point composite MACE: first confirmed event of CV death, non-fatal MI, nonfatal stroke or hospitalization for unstable angina Secondary outcome: 3-point MACE (Cv death.nonfatal MI/nonfatal stroke), fatal/nonfatal MI, fatal/nonfatal stroke, all-cause death, hospitalization for heart failure Adjudicated by independent clinical events classification committee	At screening/enrollemnt, 4-month, 8-month visits and then annual visits, the symptoms and appropriate management of hypoglycaemia were reviewed with participants. SH episodes were recorded systematically as prespecified events of clinical interest: episodes in which a participant was sufficietnyl disorientated or incapacitated as to require help. If >2 SH epsidoes between study visits, participants were required to discontinue medication.	Cox regression models Adjustments for age, sex, race, ethnicity, HbA1c, NYHA class, smoking, MI, COPD, CAD, stroke, >50% stenosis of carotid artery, atrial flutter/fibrillation, insulin, amputation, diabetic neuropathy, foot ulcer, blood pressure, heart rate, height, BMI, eGFR, randomized treatment, diabetes duration, geographical region.	SH association with primary composite CV end point aHR 1.55 (1.06–2.28), All-cause mortality aHR 1.83 (1.22–2.75) CV death aHR 1.72 (1.02–2.87)
Whitmer et al. (41)	Dementia: inpatient and outpatient databases based on ICD9-CM	Hospitalisation and ED diagnoses of hypoglycaemia using hospital/ED databases ICD9-CM	Cox proportional hazard regression models, adjusted for age, sex, race/ethnicity, education, BMI, duration of diabetes, 7-yr mean HbA1c, diabetes treatment,	History of severe hypoglycaemic episodes was associated with a greater risk of dementia: aHR 1.44 (1.25–1.66)
Yaffe et al. (42)	Dementia: hospital records indicating an admission associated with dementia or the use of prescribed dementia medications	Hospital records: severe hypos requiring admission and identified as primary or secondary diagnosis related to overnight hospitalisation. No information on milder hypos not requiring admission	Cox Proportional Hazard Regression. Adjustments for age, educational level, race/ethnicity, and any other covariates significantly associated with severe hypoglycaemia or dementia in bivariate analysis	Hypoglycaemia associated with increased risk of dementia: aHR 2.09 (1.00–4.35)
Zinman et al. (45)	The primary composite outcome in the time-to-event analysis was the first occurrence of death from cardiovascular causes, nonfatal myocardial infarction, or nonfatal stroke.	Self-reported hypoglycaemia was a secondary safety endpoint, reported using patient diaries and transcribed into case report form. SH defiend as requiring assistance of another person to administer fast-acting carbohydrates, glucagon or other resuscitative action–reported as a medical event of special interest. Confirmed hypoglycaemia defined as SH or minor hypoglycaemia (<3.1mmol/L). Nocturnal hypoglycaemia defined as episodes occurring between 00:01 and 05:59h. Patients asked to check blood glucose whenever a hypoglycaemic episode was suspected.	Cox regression to analyze time to first MACE, CV death, non-CV death or all-cause mortality with either SH at any time (yes/no) as a factors or with hypoglycaemia (SH or confirmed, yes/no) as a time-dependent covariate. Adjustments for randomized treatment, basline covariates, concomitant insulin use, HbA1c during trial, concomitant sufonylurea/glinide use, eGFR and event adjudication committee-confirmed hospitalization for heart failure during the trial (time-dependent covariates)	MACE up to one year with SH aHR 1.90 (1.30–2.90) All-cause death up to one year with SH aHR 2.70 (1.90–3.90)
Zhao et al. (43)	ICD-9-CM codes. Macrovascular: MI, stroke, congestive heart failure, peripheral vascular disease. Microvascualr: renal, ophthalmic or neurologic manifestations with diabetes.	ICD-9-CM codes	Propensity score matching (greedy 5 to 1 method) for noncomparable baseline characteristics Cox proportional hazard regression models controlling for covariates, including baseline demographic and illness characteristics, vital signs, prior medication, and index drug	HR 2.00 (1.63-2.44) for cardiovascular events, HR 1.76 (1.46, 2.11) for microvascular complications HR 1.29 (0.94, 1.77) for mortality.
Zhao et al. (44)	ICD-9-CM codes for fall-related events (fractures, head injuries) with a fall being the external cause within a two-day window.	ICD-9-CM codes	McNemar tests, generalized estimating equation (GEE) Matching on age, gender, ethnicity and medical service Adjustments for social demographic and illness characteristics, vital signs and medication use	aOR 2.70 (1.64, 4.47) for fall-related events in the hypoglycaemia group
Zoungas et al. (46)	First major macrovascular event = death from cardiovascular cause, non-fatal MI, non-fatal stroke First major microvascular event = new or worsening nephropathy or retinopathy Secondary outcomes = death from any cause and death from a cardiovascular event Independent adjudication by blinded committee	Blood glucose level <2.8 mmol/L or typical symptoms/signs without other apparent cause. Those with transient neurological dysfunction who required help from 3^rd^ party were considered to have severe hypoglycaemia. Minor hypoglycaemia if transient dysfunction of CNS and able to treat themselves.	Cox proportional-hazard models adjusted for covariates. Baseline: sex, duration of diabetes, treatment allocation, history of macrovascular or microvascular disease, ever smoker. Time dependent covariates during follow-up: age, HbA1c, body mass index, creatinine, urine albumin to creatinine ratio, systolic blood pressure, diabetes and blood pressure drugs.	HR 2.88 (2.01, 4.12) major macrovascular events, HR 1.81 (1.19, 2.74) major microvascular events, HR 2.68 (1.72, 4.19) death from cardiovascular cause, HR 2.69 (1.97, 3.67) death from any cause

OR, Odds ratio; HR, Hazard ratio; 95% CI, 95% Confidence Interval; T2DM,Type 2 diabetes mellitus; HbA1C,glycated haemoglobin; CKD, chronic kidney disease; DPP-4,dipetidyl-peptidase-4; SU, Sulfonylureas; MI, myocardial infarction; AF, atrial fibrillation; CVD, cardiovascular disease; HES, Hospital Episode Statistic; CPRD, Clinical Practice Research Datalink database; TUG, Timed Up and Go Test; GDS-15,Geriatric Depression Scale; CCI, Charlson comorbidity index; IFG, impaired fasting glucose; IGT, impaired glucose tolerance; SH, severe hypoglycaemia; ACEi, angiotension-converting enzyme inhibitor; ARB, angiotension receptor blocker; TIA, transient ischemic attack; MMSE, Mini-Mental State Examination; eGFR, estimated glomerular filtration rate.

The outcomes (adverse events) of interest were dementia, falls and fractures, composite cardiovascular (macrovascular) and microvascular events and all-cause mortality.

We independently assessed study validity by looking at how hypoglycemia and serious adverse events were recorded and whether adjustments were made for potential confounding factors.

### Data Synthesis and Analysis

We performed a random effects meta-analysis of the relative effect measures using the generic inverse variance method (Revman 5.3, Nordic Cochrane Centre, Kobenhavn). As adverse events are rare, odds ratios and risk or hazard ratios will yield similar estimates of relative effect, and we have pooled all of them using a random effects model. The random effects method for meta-analyses takes into account heterogeneity and estimates an average effect, considering differences in intervention effects as random, rather than the single true effect pooled estimate that arises from the fixed effect model.

Heterogeneity was assessed by using the I^2^ statistic and visual inspection of the forest plots.

We planned to construct a funnel plot if we had more than 10 studies in the meta-analysis (without evidence of statistical heterogeneity - I^2^ <50%).

As this was a systematic literature review, ethics approval was not required.

## Results

**Figure 1:**
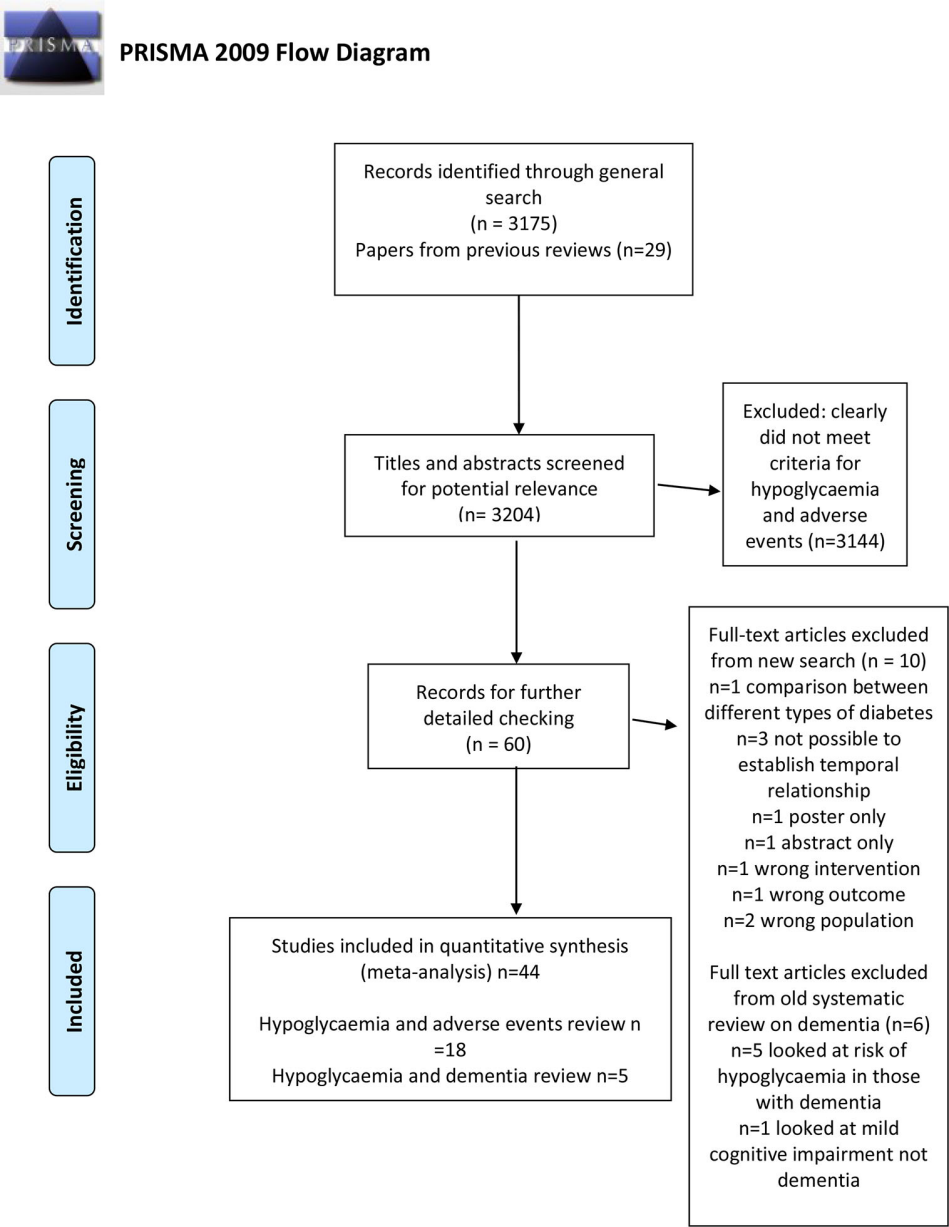
PRISMA flow chart.

We screened 3,175 citations in addition to the 29 papers that were included in the previous reviews. We included 44 studies with a total of 2,507,434 participants (3–24). The flow chart of the study selection is shown in **Figure 1**.

Characteristics of the included studies and participants are shown in **Table 1**. The included studies consist of 21 retrospective, 11 prospective and 12 post-hoc analyses. Geographical locations were diverse and included North America, Canada, Asia and Europe.

29 studies looked at patients with type 2 diabetes, whereas the remaining studies included patients with a mix of type 1 diabetes, type 2 diabetes, and impaired glucose tolerance/impaired fasting glucose. Four studies focussed on oral hypoglycaemics (37–39, 46). We report details of study validity (ascertainment of adverse outcomes, and confounding factors) in **Table 2** and summarize the key features below.

### Ascertainment of Hypoglycemia

Most of the studies relied on hospital or claims data records for severe hypoglycaemic events, ie hypoglycemia that requires help from another person to be managed/treated. 11 studies rely on either a history of self-reported hypoglycaemic episodes, questionnaires, or provided participants with diaries and glucometers (4–7, 10, 13, 16, 32, 34, 40, 45). These studies would be considered to be lower quality because of lack of medical documentation and high risk of recall bias.

### Ascertainment of Adverse Events

12 studies used pre-specified outcomes from RCTs and one non-interventional study (5, 9–11, 13, 25, 34, 36, 40, 45, 46).

29 studies measured adverse events through database or medical records codes, one study relied on a professional interviewer with questionnaire (7) and one study on self-report/GP questionnaires (4). Dementia was ascertained though a wide variety of tests.

### Confounding Factors

The included studies took account of potential confounding through the use of multivariate logistic regression models. Five studies used propensity score matching (18, 24, 34, 43, 47).

## Meta-Analysis

### Association Between Hypoglycemia and Macro- and Microvascular Disease and Cardiovascular Death

19 studies confirmed a significant association between hypoglycemia and macrovascular complications (3, 4, 10, 11, 14, 16, 17, 18, 20, 23, 26, 28, 34, 36, 38, 40, 43, 45, 46). The pooled odds ratio was 1.81 (95% CI 1.70–1.94). There was low heterogeneity (I^2^ = 6%).

**Figure 2:**
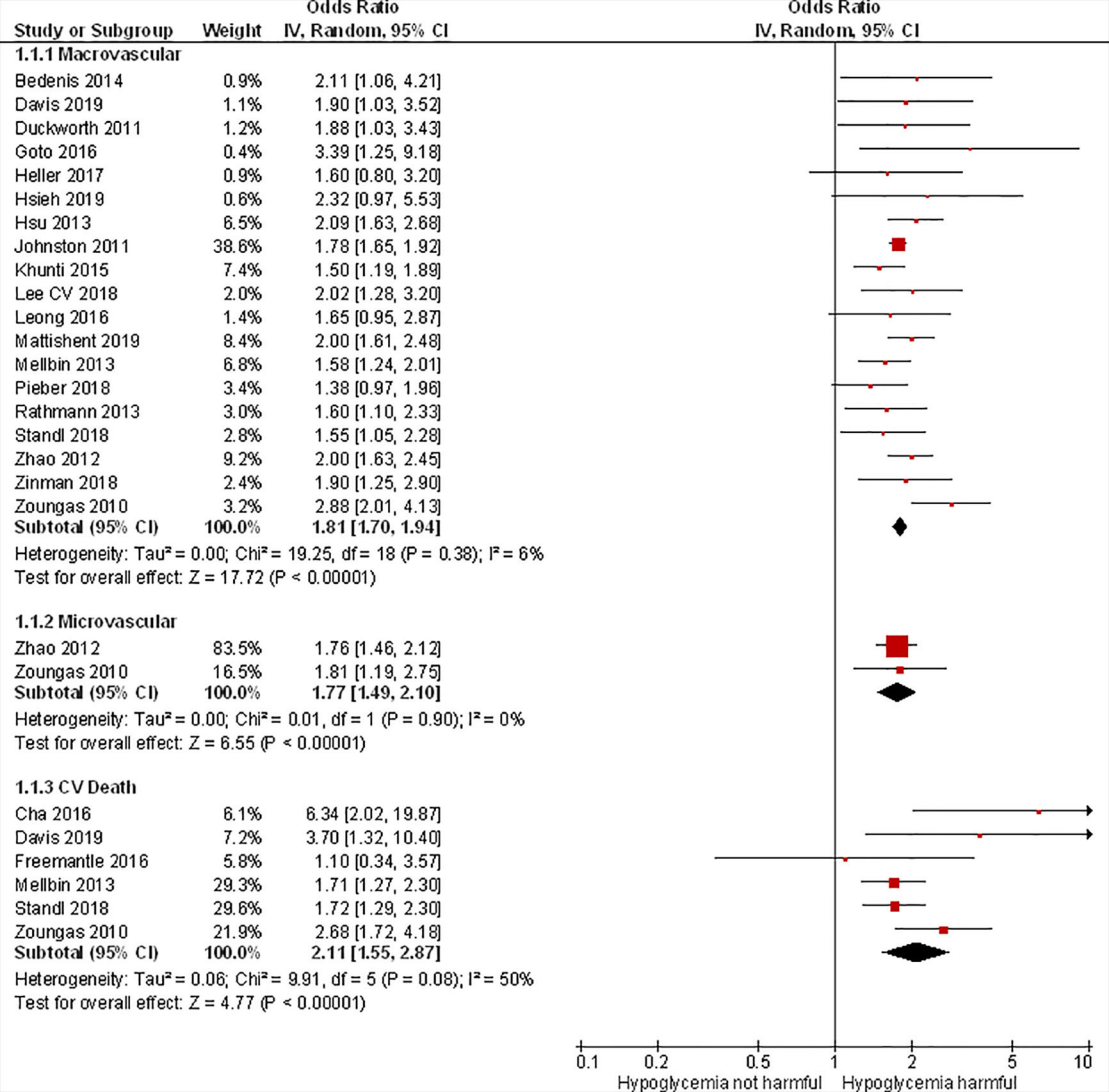
Meta-analysis of association between hypoglycemia and macro- and microvascular disease and cardiovascular death.

Similarly, two studies confirmed a significant association between hypoglycemia and microvascular complications (43, 46). The microvascular complications covered in the study were nephropathy or retinopathy (46) and a composite endpoint of several complications (43). The pooled odds ratio was 1.77 (95% CI 1.49–2.10) with no evidence of heterogeneity (I^2^ = 0%) (**Figure 2**).

Six studies reported on cardiovascular death and confirmed a significant association between hypoglycaemia and cardiovascular death. The pooled odds ratio was 2.11 (95% CI 1.55–2.87) with evidence of moderate heterogeneity (I^2^ = 50%) (6, 10, 13, 34, 40, 46).

### Association Between Hypoglycemia and Falls or Fractures

Six studies reported on falls (3, 7, 22, 30, 39, 44) with a pooled odds ratio of 1.78 (95% CI 1.44–2.21) and substantial heterogeneity (I^2^ = 87%).

**Figure 3:**
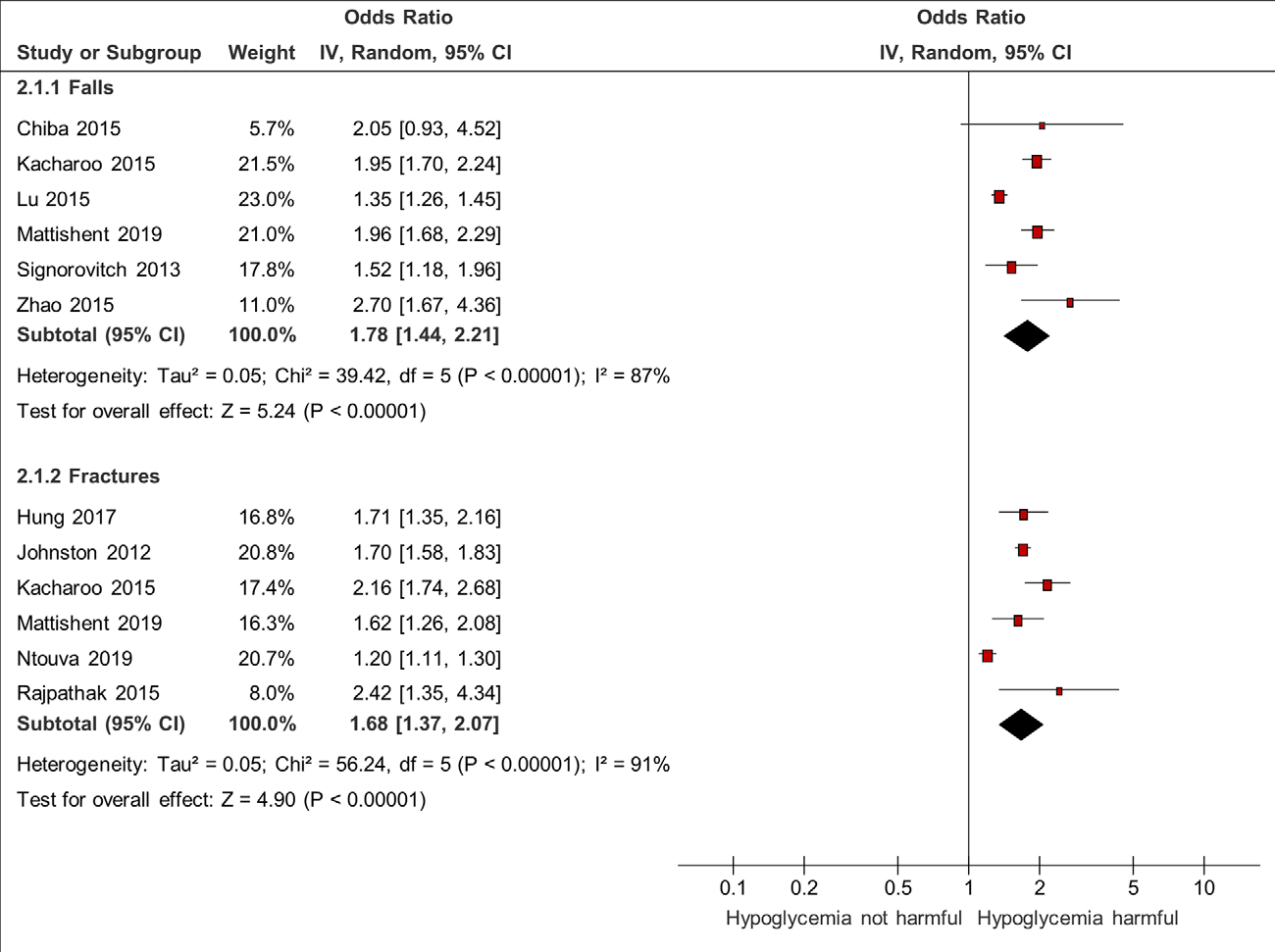
Meta-analysis of association between hypoglycemia and falls and fractures.

Six studies reported on fractures with a pooled odds ratio of 1.68 (95% CI 1.37–2.07) and considerable heterogeneity (I^2^ = 91%) (3, 19, 20, 22, 35, 37) (**Figure 3**).

### Association Between Hypoglycemia and Mortality

**Figure 4:**
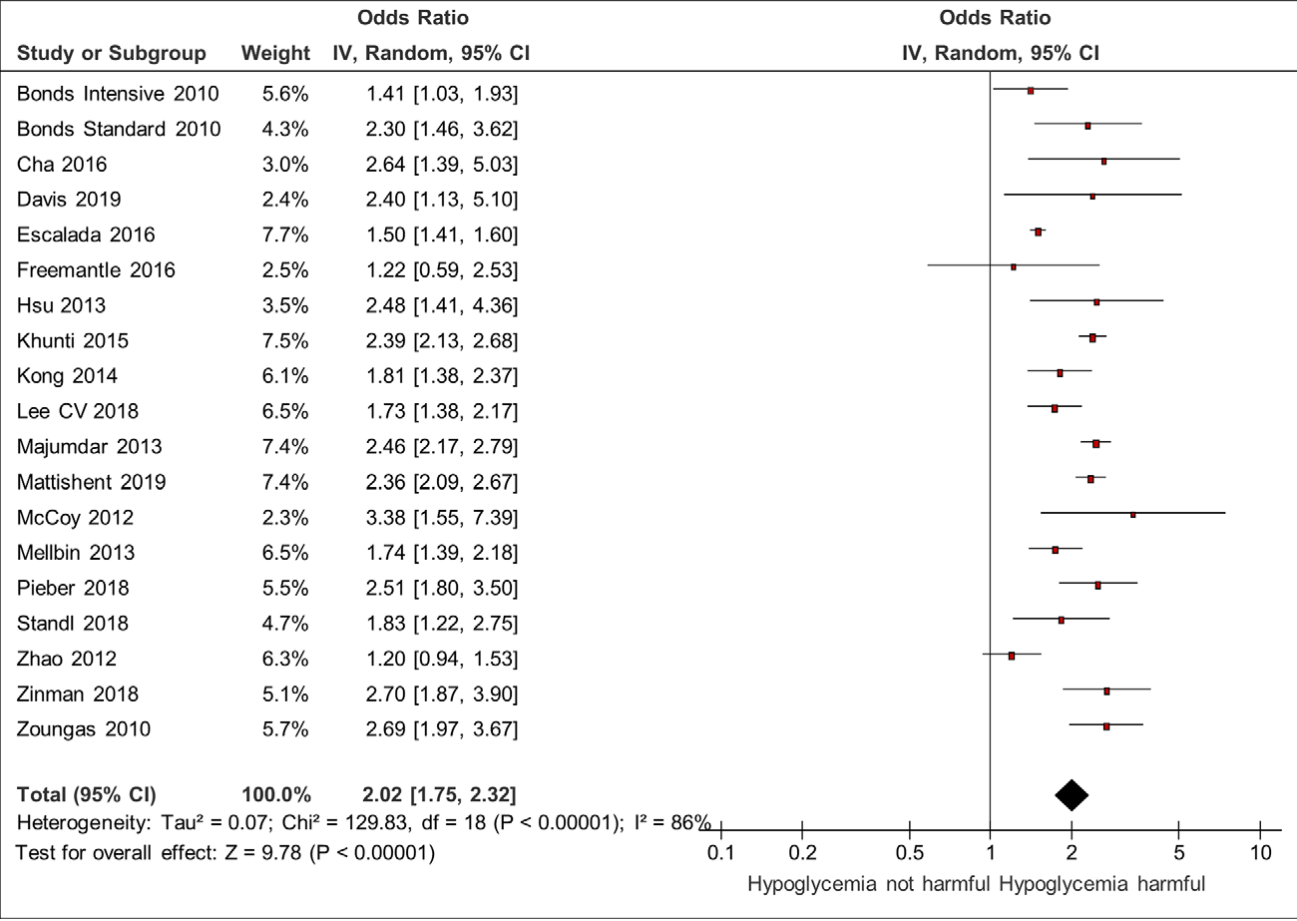
Meta-analysis of association between hypoglycemia and mortality.

18 studies reported on overall mortality confirming a significant association between hypoglycemia and mortality with a pooled odds ratio of 2.02 (95% CI 1.75–2.32) and substantial heterogeneity (I^2^ = 86%) (3, 5, 6, 10, 12, 13, 18, 23, 25, 26, 31, 32, 34, 36, 40, 43, 45, 46) (**Figure 4**). Despite the heterogeneity, the direction of association was consistent across all the studies in the Forest plot. Two studies did not find a statistically significant association between hypoglycemia and mortality (13, 43).

**Figure 5:**
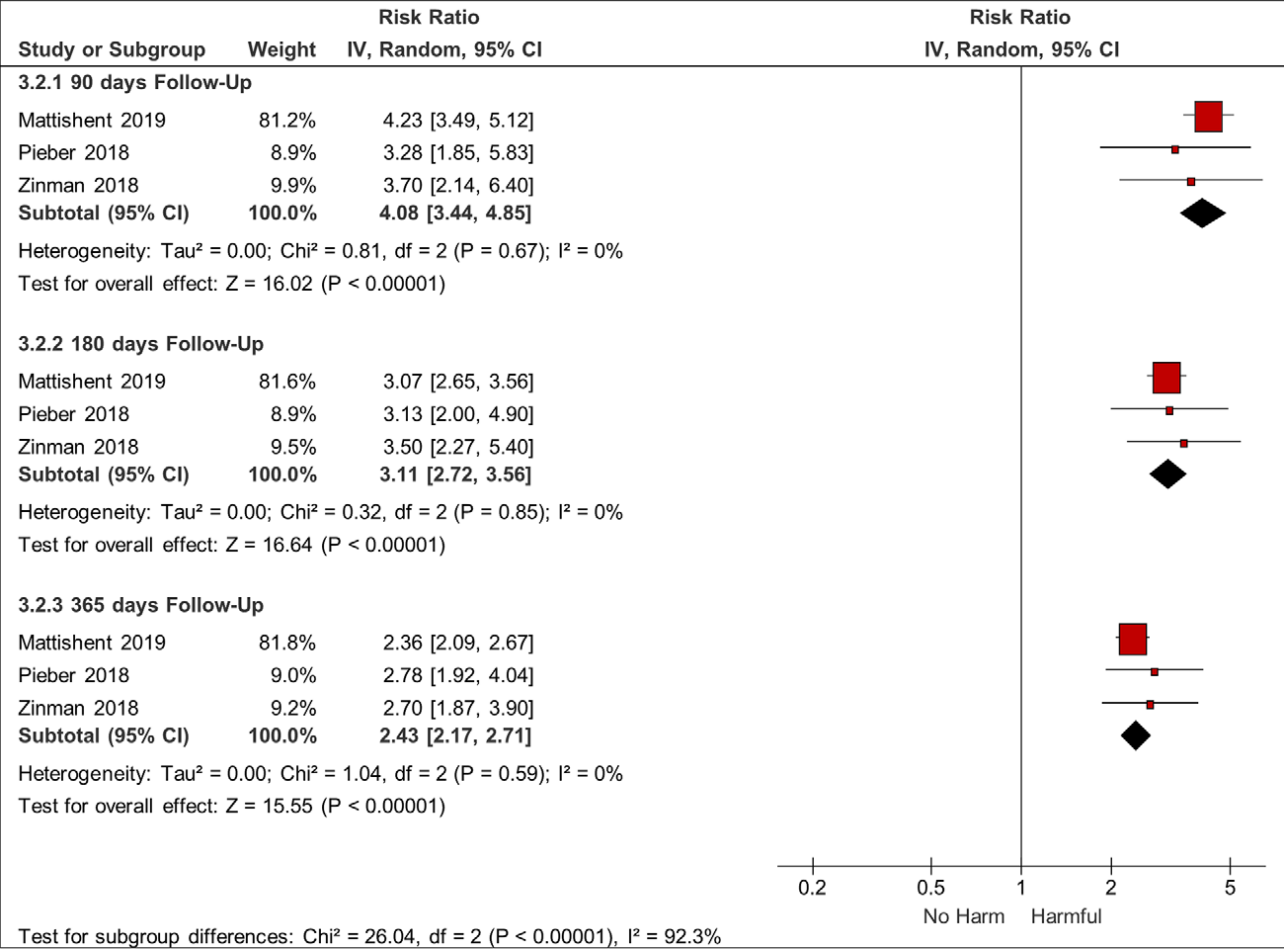
Sensitivity analysis: Mortality HRs at different durations of follow-up.

We also conducted a sensitivity analysis for mortality hazard ratios (HRs) at different durations of follow-up, which three studies addressed (**Figure 5**). The HR is greatest early on [pooled HR 4.08 (95% CI 3.44–4.85) at 90-days follow-up] and diminishes with time after the hypoglycaemic episode [pooled HR 2.43 (95% CI 2.17–2.71) at 365-days follow-up] (3, 36, 45).

### Association between hypoglycemia and dementia

**Figure 6:**
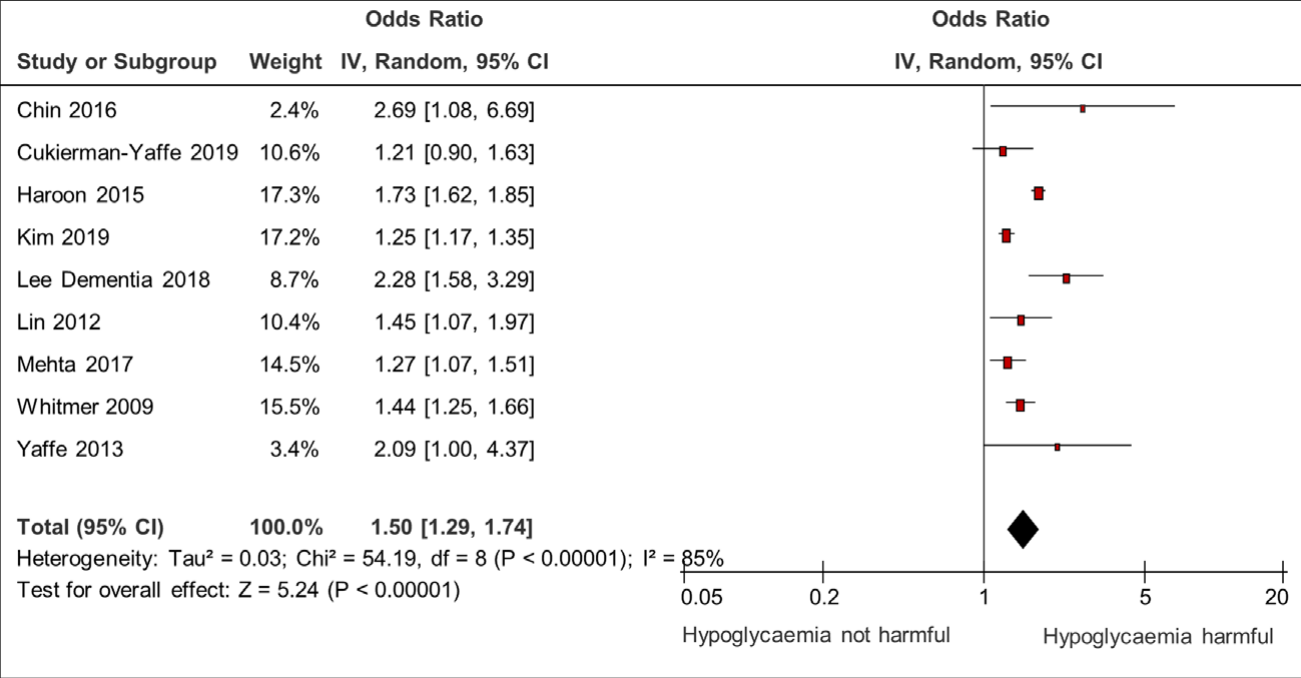
Meta-analysis of association between hypoglycemia and dementia.

9 studies confirmed a significant association between hypoglycemia and dementia (8, 9, 15, 24, 27, 33, 41, 42) with a pooled odds ratio of 1.50 (95% CI 1.29–1.74). There was substantial heterogeneity with I^2^ = 85% (**Figure 6**).

### Sensitivity analysis – restriction to studies with patients’ mean age ≥65 years

As there is no consensus on the age-threshold for ‘older’ patients, we conducted a sensitivity analysis where we excluded studies in younger patients (mean age< 65 years). The pooled odds ratios were modestly increased when restricted to older patients, thus suggesting the possibility of greater harm associated with hypoglycemia with older age: Macrovascular events OR 1.88 (95% CI 1.72–2.07), Falls OR 1.98 (95% CI 1.80–2.19), and Death OR 2.25 (95% CI 1.78–2.83).

### Publication Bias and Selective Outcome Reporting

We constructed a funnel plot for the meta-analysis on the association between hypoglycemia and vascular events, as more than ten studies were included in the analysis (see **Supplemental Figure 1**). On visual inspection of the funnel plot there are very few small studies that contributed to the meta-analysis, and it is difficult to judge presence or absence of asymmetry. As such, we cannot rule out the possibility of selective reporting or publication bias.

## Discussion

This updated meta-analysis incorporated 23 new studies to the previously published meta-analyses on the association between hypoglycemia and adverse events in older people with diabetes. The total of 44 observational studies (involving over 2.5 million participants) confirms the danger of hypoglycemia in older people with diabetes, which is consistent with the previously published reviews.

Our meta-analyses demonstrated significant associations between hypoglycemia and death, dementia, macrovascular and microvascular complications, cardiovascular death, and falls and fractures. In our previous work, we were unable to report on cardiovascular death due to lack of data, but we have now been able to perform this meta-analysis with six studies (6 10, 13, 34, 40, 46).

In addition, our new sensitivity analysis of mortality HRs at different times of follow-up also shows that the HR for death is greatest early on and diminishes with longer follow-up duration. This suggests that the association between hypoglycaemic episodes and death is not just due to baseline confounding. If that were the case, we would not expect to see diminishing hazard ratios with longer intervals of time after the hypoglycaemic episode.

The collated evidence regarding serious harm supports our argument that we should prioritize the avoidance of hypoglycemia in this vulnerable group of patients.

An international consensus on clinical targets for continuous glucose monitoring data was published in June 2019, highlighted that older adults with diabetes should spend less than 15 minutes per day in the hypoglycaemic range (<3.9mmol/L) (48). This is of particular significance, as hypoglycaemic episodes are often missed in older people with diabetes (49).

We are aware of studies that have identified potential links between hypoglycemia and cardiac arrhythmias (50, 51), which could account in part for the findings of increased risk of myocardial infarction, stroke, falls and death following hypoglycemia.

Regarding cognitive adverse effects, a recent study found that hypoglycemia was associated with smaller total brain volume on MRI (27). Furthermore, Gibas et al. put forward the theory of “brain starvation” in patients with type 2 diabetes, due to concurrent hyperinsulinemia and relative hypoglycemia due to insulin resistance resulting in apoptosis of healthy neurons from catabolic degeneration (52). Radiological studies in patient who had suffered profound hypoglycemia have shown that neurons in the hippocampal and temporal areas, cerebral cortex, substantia nigra, and basal ganglia are particularly sensitive to hypoglycemia (53). Cognitive decline may in turn pre-dispose older frail people to falls, fractures and death following hypoglycemia.

### Limitations

We cannot prove causality due to the observational nature of the studies. Although we identified heterogeneity in some of the meta-analyses, the direction of the effect was consistent amongst the studies. Factors which could be influencing heterogeneity include different classes of medications, different geographical locations, different study designs and the accuracy of ascertainment of hypoglycaemic episodes and adverse events.

We considered summarizing the evidence using GRADE, however, this tool is mainly designed for recommendations on healthcare intervention and not for etiology and prognostic studies. The two main areas within GRADE that cannot be applied here are ‘measure of indirectness’ and ‘estimation of absolute effect size’.

Regarding publication bias, if null or negative findings are not fully reported, this may result in inflated estimates of association in the meta-analyses. However, there were very few small studies in the funnel plot analysis we performed in relation to cardiovascular events, and so it is not possible for us to rule out bias from non-publication of small studies.

## Conclusions

Our updated review provides a strong evidence base to support and strengthen our argument about the importance of adopting a hypoglycemia minimization strategy. Further work has to be carried out in older people with diabetes to establish effective hypoglycemia minimization strategies through better monitoring *via* continuous glucose monitoring coupled with de-intensification of management regimes, rather than the pursuit of specific HbA1c targets.

## Author Contributions

KM and YKL designed the systematic review and meta-analysis, carried out the data extraction, analysis and drafted the manuscript. YKL is the guarantor. All authors contributed to the article and approved the submitted version.

## Conflict of Interest

The authors declare that the research was conducted in the absence of any commercial or financial relationships that could be construed as a potential conflict of interest.
