# Supplementary figures and images for: Meta-Analysis: Association Between Hypoglycemia and Serious Adverse Events in Older Patients Treated With Glucose-Lowering Agents
Source: Front Endocrinol (Lausanne). 2021 Mar 8;12:571568. doi: 10.3389/fendo.2021.571568 (PMC7982741; doi:10.3389/fendo.2021.571568)

**Supplemental Figure 1: Funnel Plot**

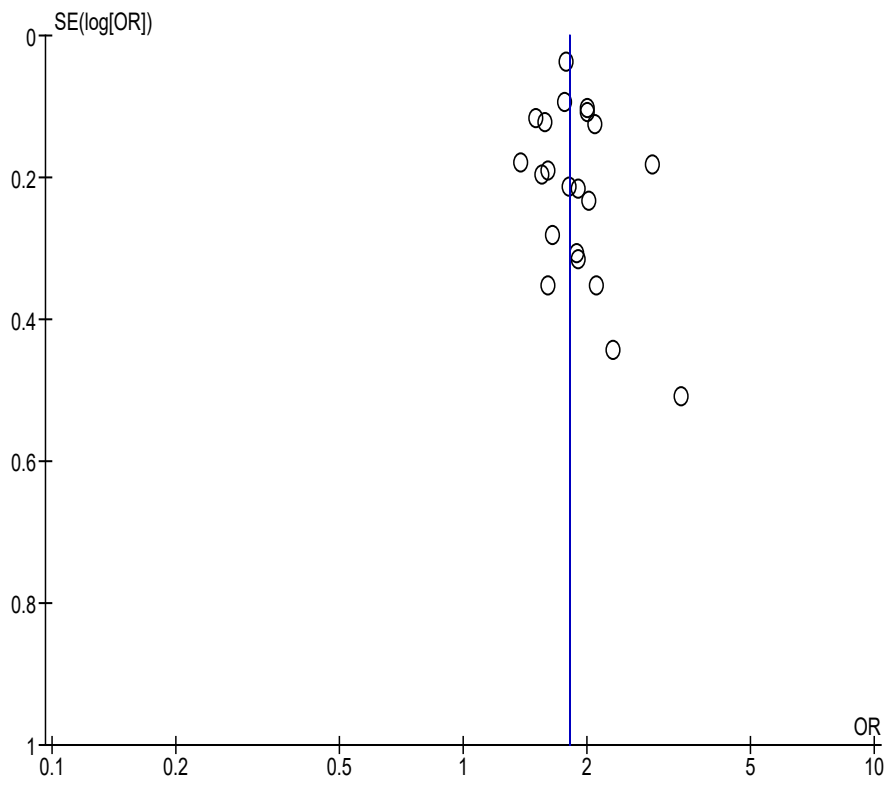

Supplement: Supplementary file 1 [file Image_1.pdf]
