# Supplementary material for: Meta-Analysis: Association Between Hypoglycemia and Serious Adverse Events in Older Patients Treated With Glucose-Lowering Agents
Source: Front Endocrinol (Lausanne). 2021 Mar 8;12:571568. doi: 10.3389/fendo.2021.571568 (PMC7982741; doi:10.3389/fendo.2021.571568)
Supplement: Supplementary file 2 [file DataSheet_1.docx]

**Appendix: Search strategies**

**Ovid SP**

| 1. | diabetes-mellitus.mp. [mp=title, abstract, subject headings, heading word, drug trade name, original title, device manufacturer, drug manufacturer, device trade name, keyword] |  |  |  |
| --- | --- | --- | --- | --- |
| 2. | older-patient?.mp. [mp=title, abstract, subject headings, heading word, drug trade name, original title, device manufacturer, drug manufacturer, device trade name, keyword] |  |  |  |
| 3. | older-adult?.mp. [mp=title, abstract, subject headings, heading word, drug trade name, original title, device manufacturer, drug manufacturer, device trade name, keyword] |  |  |  |
| 4. | elderly.mp. [mp=title, abstract, subject headings, heading word, drug trade name, original title, device manufacturer, drug manufacturer, device trade name, keyword] |  |  |  |
| 5. | geriatric.mp. [mp=title, abstract, subject headings, heading word, drug trade name, original title, device manufacturer, drug manufacturer, device trade name, keyword] |  |  |  |
| 6. | veterans.mp. [mp=title, abstract, subject headings, heading word, drug trade name, original title, device manufacturer, drug manufacturer, device trade name, keyword] |  |  |  |
| 7. | hypoglyc?emia.mp. [mp=title, abstract, subject headings, heading word, drug trade name, original title, device manufacturer, drug manufacturer, device trade name, keyword] |  |  |  |
| 8. | 2 or 3 or 4 or 5 or 6 |  |  |  |
| 9. | 1 and 7 and 8 |  |  |  |
| 10. | limit 9 to (english and last 10 years) |  |  |  |

**PubMed update**

((("diabetes mellitus"[MeSH Terms] OR ("diabetes"[All Fields] AND "mellitus"[All Fields]) OR "diabetes mellitus"[All Fields]) AND ("hypoglycaemia"[All Fields] OR "hypoglycemia"[MeSH Terms] OR "hypoglycemia"[All Fields]) AND (("aged"[MeSH Terms] OR "aged"[All Fields] OR ("older"[All Fields] AND "adult"[All Fields]) OR "older adult"[All Fields]) OR ("aged"[MeSH Terms] OR "aged"[All Fields] OR "elderly"[All Fields]) OR geriatric[All Fields] OR ("veterans"[MeSH Terms] OR "veterans"[All Fields]) OR older-patient?[All Fields]))

Updated search

**PubMed**

("diabetes mellitus"[MeSH Terms] OR ("diabetes"[All Fields] AND "mellitus"[All Fields]) OR "diabetes mellitus"[All Fields]) AND ("older patients"[All Fields] OR "older patient"[All Fields] OR "elderly"[All Fields] OR geriatric?[All Fields] OR "veterans"[MeSH Terms] OR "Aged"[MeSH Terms] OR "veterans"[All Fields] OR "older adults"[All Fields]) AND ("hypoglycaemia"[All Fields] OR "hypoglycemia"[MeSH Terms] OR "hypoglycemia"[All Fields]) AND English[lang]).
